# Leading Comorbidity associated with 30-day post-anesthetic mortality in geriatric surgical patients in Taiwan: a retrospective study from the health insurance data

**DOI:** 10.1186/s12877-017-0629-7

**Published:** 2017-10-24

**Authors:** Chun-Lin Chu, Hung-Yi Chiou, Wei-Han Chou, Po-Ya Chang, Yi-You Huang, Huei-Ming Yeh

**Affiliations:** 1 Institute of Biomedical Engineering National Taiwan University, No1, Sec 1, Jen-Ai Rd., Taipei, Taiwan; 2 Department of Anesthesiology, National Taiwan University Hospital Yun-Lin Branch, No 579, Sec 2, Yun-Lin Rd., Douliu, Yun-Lin Taiwan; 3 School of Public Health, Taipei Medical University, No 250, Wu-Shin Street, Taipei, Taiwan; 4 Department of Anesthesiology, National Taiwan University Hospital, No 7 Chung-Shan South Road, Taipei, Taiwan, Republic of China

**Keywords:** Comorbidity, Post-anesthesia mortality

## Abstract

**Background:**

Elderly patients with aged physical status and increased underlying disease suffered from more postoperative complication and mortality. We design this retrospective cohort study to investigate the relationship between existing comorbidity of elder patients and 30 day post-anesthetic mortality by using International Classification of Diseases, 9th Revision, Clinical Modification (ICD-9-CM) from Health Insurance Database.

**Methods:**

Patients aged above 65 years old who received anesthesia between 2000 and 2010 were included from 1 million Longitudinal Health Insurance Database in (LHID) 2005 in Taiwan. We use age, sex, type of surgery to calculate propensity score and match death group and survival one with 1:4 ratio (death: survival = 1401: 5823). Multivariate logistic model with stepwise variable selection was employed to investigate the factors affecting death 30 days after anesthesia.

**Results:**

Thirty seven comorbidities can independently predict the post-anesthetic mortality. In our study, the leading comorbidities predict post-anesthetic mortality is chronic renal disease (OR = 2.806), acute myocardial infarction (OR = 4.58), and intracranial hemorrhage (OR = 3.758).

**Conclusions:**

In this study, we present the leading comorbidity contributing to the postoperative mortality in elderly patients in Taiwan from National Health Insurance Database. Chronic renal failure is the leading contributing comorbidity of 30 days mortality after anesthesia in Taiwan which can be explained by the great number of hemodialysis and prolong life span under National Taiwan Health Insurance. Large scale database can offer enormous information which can help to improve quality of medical care.

**Electronic supplementary material:**

The online version of this article (10.1186/s12877-017-0629-7) contains supplementary material, which is available to authorized users.

## Background

Increased life expectancy, improvement of anesthesia safety and less invasive surgical techniques have made greater number of geriatric patients receive surgical intervention. With aged physical status and increased underlying disease, the risk of anesthesia and postoperative complication and mortality is much higher than other populations [1, 2].

The main four factors of surgical risk and outcome in patients older than 65 years old are age,physiologic status,coexisting disease, and type of procedure [3, 4]. Earlier studies suggest that anesthetic complications are related to age and some studies also have corroborated an association of mortality and morbidity with American Society of Anesthesiologists physical status (ASA-PS) scores. The surgical procedure itself significantly influence postoperative risk and it can be classified to low, intermediate, and high-risk surgery [5].

The ASA-PS classification introduced to clinical practice since 1941 was used worldwide to quantify the amount of physiological reserve that a patient possesses when assessed before a surgical procedure. This classification is validated as a reliable independent predictor of medical complications and mortality following surgery in peer review articles [6, 7]. However, the ASA-PS scale has unreliability due to its inherent subjectivity which resulted in different ASA class rated in one patient by different anesthesiologists [8]. It is useful but lack of scientific precision.

To date, national health insurance database in Taiwan has recruited most patients’ information and medical record for more than 10 years. Several studies have been published by using the reimbursement claims data of Taiwan’s national Health Insurance [9–11]. We design this retrospective cohort study to investigate the relationship of existing comorbidity of geriatric patients who came for anesthesia with 30 day post-anesthetic mortality rate by using International Classification of Diseases, 9th Revision, Clinical Modification (ICD-9-CM). We hope to investigate the impact of different underlying comorbidity of the geriatric patient on post-anesthesia mortality.

## Methods

### Data base

Taiwan’s National Health Insurance was put into practice since 1995 and covered more than 22.6 million residents in Taiwan. Taiwan’s National Health Research Institutes established a National Health Research Database which record all in-patient and out-patient medical services for research [9]. This study used the 1 million Longitudinal Health Insurance Database in 2005 (LHID), which means 1 million patients were randomly enrolled in 2005 and the longitudinal database included all the issue from 2000 to 2010. The database was decoded with patient identifications to protect patients’ privacy and scrambled for further public access. This study was approved by National Taiwan University Hospital Ethics Committee (201411078RINC) and inform consent was waived.

### Study sample

**Fig. 1:**
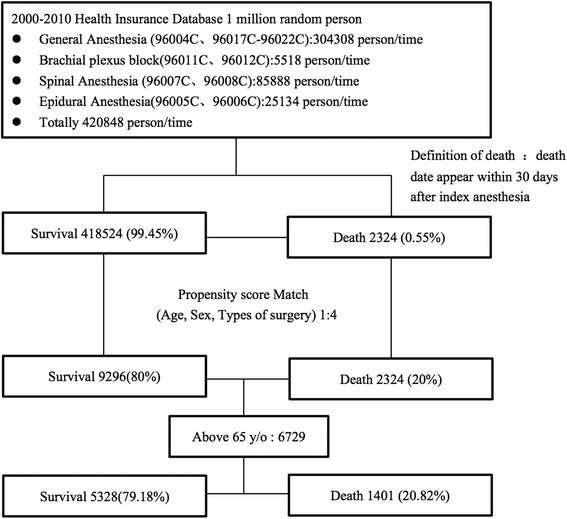
Flow chart of study design

The study sample is the patients aged above 65 years old and received anesthesia between 2000 and 2010. There were 420,848 index surgery requiring anesthesia in this period, including general anesthesia 304,308 times, brachial plexus block 5518 times, spinal anesthesia 85,888 times, and epidural anesthesia 2,5134 times. We defined mortality as death date appeared within 30 days after index surgery whether in hospital or not. There were 2324 death and 418,524 survival after index surgery. Due to tremendous difference in population, we use age, sex, type of surgery to calculate propensity score [12, 13] and match death and survival group with 1:4. Among them, there were 6729 patients aged above 65 years old and 1401 patients were dead (Fig. 1).

### Key variable of interest

We use International Classification of Diseases, 9th Revision, Clinical Modification (ICD-9-CM) appeared 2 years before index surgery in our database as comorbidity. The definition of comorbidity means the patient was diagnosed for more than 3 times and the interval was more than 28 days which including ischemic heart disease, hypertension, heart failure, vascular disease, respiratory disease, disease of liver and biliary tract, disease of GI system, urinary disease, endocrine disease, musculoskeletal disease, infectious disease, CVA or trauma, cancer, other disease..(Additional file 1) Due to disease categorization is complex, we therefore aggregated codes into disease group to resemble clinical pre-anesthetic usage. This process was conducted independently by three anesthesiologists.

### Statistical analysis

The difference of comorbidity in death and survival group 30 days after index surgery was analyzed by Chi-Square test. We use conditional logistic regression to correct age, gender, type of surgery and other comorbidity, then analysis the correlation of comorbidity with death. Multivariate logistic model with stepwise variable selection [14] was employed to investigate the factors affecting death 30 days after anesthesia. We perform calculation by SAS statistical package (SAS System for Windows, Version 9.3; SAS Institute Inc., Cary, NC).

## Results

More than one hundred codes were given out when we count all the comorbidity ICD-9 code in death group. Seventy three codes were selected after aggregation by expertise. (Additional file 1) All the comorbidity was compared by chi square test under 1:4 ratio by matching age, sex, type of surgery as Table 1 listed. Age and sex were both statistically significant after propensity score matching. The crude odds ratio and adjusted odds of each comorbidity (Table 2) was counted and then 37 leading comorbidities (Table 3) which can independently predict 30 days post-anesthetic mortality in geriatric patients were ranked by multivariate logistic model with stepwise variable selection. In our study, the leading comorbidities predict post-anesthetic mortality is chronic renal disease, acute myocardial infarction, and intracerebral hemorrhage.

**Table 1:** Correlation analysis of comorbidity and mortality in more than 65-year-old patients, *N* = 6729 (match 1:4)

Comorbidity	Non-Death (*N* = 5823)		Death (*N* = 1401)		*P*
	n	%	n	%	
Age(mean,sd)	76.72	7.08	78.08	7.41	<.0001
Sex(Male)	3563	66.87	855	61.03	<.0001
Ischemic heart disease					
Acute myocardial infarction	66	1.24	68	4.85	<.0001
Coronary atherosclerosis of native coronary artery	661	12.41	251	17.92	<.0001
Hypertension	1556	29.2	616	43.97	<.0001
Heart failure					
Heart failure	232	4.35	155	11.06	<.0001
Cardiogenic shock	106	1.99	66	4.71	<.0001
Vascular disease					
Arterial embolism and thrombosis of lower extremity	39	0.73	26	1.86	0.0002
Gangrene	106	1.99	66	4.71	<.0001
Respiratory disease					
Pneumonia, organism unspecified	339	6.36	165	11.78	<.0001
Pneumonitis due to inhalation of food or vomitus	61	1.14	18	1.28	0.7694
Empyema, without mention of fistula	13	0.24	9	0.64	0.0393
Chronic bronchitis	471	8.84	162	11.56	0.0022
Pleurisy, unspecified pleural effusion	27	0.51	12	0.86	0.1812
Pulmonary insufficiency following trauma and surgery	263	4.94	88	6.28	0.0515
Disease of liver and biliary tract					
Chronic liver disease and cirrhosis	260	4.88	97	6.92	0.003
Disease of GI system					
Gastric ulcer, chronic or unspecified with hemorrhage	303	5.69	141	10.06	<.0001
Acute vascular insufficiency of intestine	21	0.39	30	2.14	<.0001
Intestinal or peritoneal adhesions with obstruction	133	2.5	52	3.71	0.0171
Hemorrhage of gastrointestinal tract	77	1.45	64	4.57	<.0001
Gastric ulcer,chronic or unspecified with perforation	303	5.69	141	10.06	<.0001
Duodenal ulcer, chronic or unspecified with perforation	164	3.08	93	6.64	<.0001
Peptic ulcer, site unspecified, chronic or unspecified with perforation	482	9.05	181	12.92	<.0001
Acute appendicitis, with generalized peritonitis	60	1.13	11	0.79	0.3348
Peritonitis	9	0.17	20	1.43	<.0001
Perforation of intestine	59	1.11	37	2.64	<.0001
Urinary disease					
Tuberculosis of ureter, tubercle bacilli found	52	0.98	48	3.43	<.0001
Unspecified hypertensive renal disease with renal failure	58	1.09	27	1.93	0.0180
Acute renal failure	50	0.94	28	2	0.0016
Chronic renal failure	206	3.87	159	11.35	<.0001
Hydronephrosis	31	0.58	9	0.64	0.9465
Calculus of ureter	227	4.26	27	1.93	<.0001
Urinary tract infection, site not specified	662	12.42	239	17.06	<.0001
Hypertrophy (benign) of prostate	948	17.79	214	15.27	0.0293
Endocrine disease	845	15.86	377	26.91	<.0001
Musculoskeletal disease					
Decubitus ulcer	94	1.76	50	3.57	<.0001
Spinal stenosis, lumbar region	1091	20.48	329	23.48	0.0156
Pathologic fracture of vertebrae	334	6.27	101	7.21	0.2253
Fracture of intertrochanteric section of femur	388	7.28	157	11.21	<.0001
Infectious disease					
Unspecified septicemia	62	1.16	12	0.86	0.4026
Necrotizing fasciitis	93	1.75	42	3	0.0041
Bacteremia	41	0.77	12	0.86	0.8744
CVA or trauma					
Obstructive hydrocephalus	91	1.71	28	2	0.5349
Other conditions of brain	16	0.3	9	0.64	0.1039
Subarachnoid hemorrhage	16	0.3	25	1.78	<.0001
Intracerebral hemorrhage	106	1.99	82	5.85	<.0001
Subdural hemorrhage	70	1.31	14	1	0.4189
Unspecified cerebral artery occlusion with cerebral infarction	382	7.17	170	12.13	<.0001
Other shock without mention of trauma	106	1.99	66	4.71	<.0001
Other and unspecified cerebral laceration	24	0.45	16	1.14	0.0051
Subarachnoid hemorrhage following injury	128	2.4	84	6	<.0001
Other and unspecified intracranial hemorrhage	20	0.38	13	0.93	0.0155
Fracture of vault of skull, closed	6	0.11	6	0.43	0.0327
Fracture of base of skull, closed	11	0.21	12	0.86	0.0006
Cancer					
Malignant neoplasm of tongue, unspecified	10	0.19	1	0.07	0.5570
Malignant neoplasm of cheek mucosa	19	0.36	7	0.5	0.5989
Malignant neoplasm of nasopharynx, unspecified	8	0.15	3	0.21	0.8761
Malignant neoplasm of hypopharynx, unspecified	8	0.15	1	0.07	0.7588
Malignant neoplasm of upper third of esophagus	12	0.23	9	0.64	0.0263
Malignant neoplasm of pyloric antrum of stomach	66	1.24	29	2.07	0.0265
Malignant neoplasm of sigmoid colon	175	3.28	52	3.71	0.4810
Malignant neoplasm of recto sigmoid junction	125	2.35	33	2.36	1.0000
Malignant neoplasm of liver, primary	59	1.11	43	3.07	<.0001
Malignant neoplasm of head of pancreas	16	0.3	13	0.93	0.0031
Malignant neoplasm of upper lobe, bronchus or lung	52	0.98	48	3.43	<.0001
Malignant neoplasm of female breast, unspecified	47	0.88	5	0.36	0.0678
Malignant neoplasm of cervix uteri, unspecified	29	0.54	4	0.29	0.3082
Malignant neoplasm of ovary	2	0.04	3	0.21	0.1079
Malignant neoplasm of prostate	103	1.93	23	1.64	0.5449
Malignant neoplasm of bladder, part unspecified	131	2.46	33	2.36	0.9000
Secondary and unspecified malignant neoplasm of lymph nodes of head, face	6	0.11	2	0.14	1.0000
Secondary malignant neoplasm of lung	21	0.39	11	0.79	0.0940
Secondary malignant neoplasm of skin	26	0.49	25	1.78	<.0001
Other diseases					
Encounter for chemotherapy	48	0.9	42	3	<.0001
Mechanical complication of other vascular device, implant and graft	150	2.82	55	3.93	0.0390

**Table 2:** Univariate and multivariate analysis of comorbidity and morality in more than 65-year-old patients, *N* = 6729 (match 1:4)

Comorbidity	Crude Odds ratio			Adjusted Odds ratio^a^		
	OR	(95%CI)		OR	(95%CI)	
Age(mean,sd)	1.026	1.018	1.035	1.024	1.015	1.034
Ischemic heart disease						
Acute myocardial infarction	4.067	2.883	5.737	4.503	3.060	6.627
Hypertension	1.902	1.686	2.147	1.406	1.223	1.616
Heart failure						
Heart failure	2.732	2.209	3.38	1.800	1.404	2.309
Cardiogenic shock	2.436	1.781	3.331	1.894	1.333	2.690
Vascular disease						
Arterial embolism and thrombosis of lower extremity	2.564	1.556	4.227	1.988	1.145	3.45
Respiratory disease						
Pneumonia, organism unspecified	1.965	1.615	2.39	1.448	1.14	1.838
Empyema, without mention of fistula	2.643	1.128	6.197	3.272	1.307	8.194
Disease of GI system						
Gastric ulcer, chronic or unspecified with hemorrhage	1.856	1.506	2.288	1.381	1.079	1.768
Acute vascular insufficiency of intestine	5.528	3.155	9.685	6.225	3.382	11.457
Hemorrhage of gastrointestinal tract	3.264	2.331	4.572	1.868	1.259	2.772
Duodenal ulcer, chronic or unspecified with perforation	2.239	1.724	2.908	2.209	1.637	2.982
Peritonitis	8.559	3.889	18.838	8.855	3.653	21.47
Perforation of intestine	2.423	1.599	3.67	2.636	1.67	4.162
Urinary disease						
Tuberculosis of ureter, tubercle bacilli found	3.6	2.421	5.353	3.699	2.347	5.831
Chronic renal failure	3.183	2.565	3.95	2.931	2.241	3.834
Calculus of ureter	0.442	0.295	0.661	0.588	0.376	0.919
Hypertrophy (benign) of prostate	0.833	0.709	0.979	0.764	0.628	0.928
Endocrine disease	0.512	0.445	0.588	0.668	0.568	0.785
Musculoskeletal disease						
Fracture of intertrochanteric section of femur, closed	1.607	1.321	1.954	1.284	1.023	1.613
Infectious disease						
Necrotizing fasciitis	1.74	1.203	2.517	1.580	1.041	2.397
CVA or trauma						
Subarachnoid hemorrhage	6.027	3.209	11.318	8.935	4.612	17.312
Intracerebral hemorrhage	3.063	2.281	4.112	3.893	2.803	5.408
Subdural hemorrhage	0.758	0.426	1.35	0.464	0.237	0.906
Unspecified cerebral artery occlusion with cerebral infarction	1.788	1.477	2.165	1.512	1.216	1.881
Other and unspecified cerebral laceration	2.553	1.353	4.819	3.058	1.513	6.178
Subarachnoid hemorrhage following injury	2.591	1.955	3.434	4.12	3.014	5.632
Fracture of vault of skull, closed	3.815	1.229	11.847	5.197	1.521	17.755
Fracture of base of skull, closed	4.176	1.839	9.484	6.424	2.666	15.478
Cancer						
Malignant neoplasm of upper third of esophagus	2.87	1.207	6.824	3.624	1.394	9.422
Malignant neoplasm of pyloric antrum of stomach	1.687	1.086	2.621	2.045	1.251	3.341
Malignant neoplasm of liver, primary	2.828	1.9	4.208	2.944	1.826	4.745
Malignant neoplasm of head of pancreas	3.109	1.492	6.478	4.035	1.809	9.002
Malignant neoplasm of female breast, unspecifie	0.402	0.16	1.014	0.335	0.119	0.939
Secondary malignant neoplasm of skin	3.705	2.133	6.436	3.418	1.796	6.506
Other diseases						
Encounter for chemotherapy	3.4	2.237	5.166	2.566	1.531	4.301

^a^Adjusted variables including age, gender, types of surgery, comorbidity

**Table 3:** Predictors of mortality in more than 65-year-old patients, *N* = 6729 (By stepwise)

Comorbidity	step	Adjusted Odds ratio		
		OR	(95%CI)	
Chronic renal failure	1	2.806	2.205	3.571
Acute myocardial infarction	2	4.58	3.135	6.691
Intracerebral hemorrhage	3	3.758	2.724	5.184
Subarachnoid hemorrhage following injury	4	3.937	2.891	5.363
Tuberculosis of ureter, tubercle bacilli found	5	3.573	2.282	5.594
Heart failure	6	1.863	1.463	2.371
Subarachnoid hemorrhage	7	8.654	4.473	16.742
Duodenal ulcer, chronic or unspecified with perforation	8	2.262	1.688	3.033
Acute vascular insufficiency of intestine	9	6.406	3.503	11.716
Peritonitis	10	9.242	3.872	22.063
Endocrine disease	11	0.656	0.559	0.768
Age(mean,sd)	12	1.024	1.015	1.034
Malignant neoplasm of liver	13	3.193	2.042	4.992
Encounter for chemotherapy	14	2.739	1.667	4.501
Perforation of intestine	15	2.683	1.705	4.222
Cardiogenic shock	16	1.963	1.388	2.776
Fracture of base of skull, closed with subarchn	17	6.619	2.812	15.58
Sex(Male)	18	0.762	0.659	0.881
Unspecified cerebral artery occlusion with cerebral infarction	19	1.514	1.221	1.877
Hemorrhage of gastrointestinal tract	20	1.903	1.291	2.805
Secondary malignant neoplasm of skin	21	3.328	1.787	6.199
Malignant neoplasm of head of pancreas	22	3.89	1.753	8.633
Malignant neoplasm of pyloric antrum of stomach	23	2.035	1.253	3.304
Pneumonia, organism unspecified	24	1.397	1.115	1.751
Other and unspecified cerebral laceration	25	3.051	1.524	6.109
Hypertrophy (benign) of prostate	26	0.78	0.645	0.943
Gastric ulcer, chronic or unspecified with hemorrhage	27	1.403	1.103	1.784
Fracture of vault of skull, closed	28	4.976	1.451	17.06
Malignant neoplasm of upper third of esophagus	29	3.391	1.315	8.742
Empyema, without mention of fistula	30	3.22	1.297	7.997
Arterial embolism and thrombosis of lower extremity	31	1.952	1.122	3.394
Malignant neoplasm of female breast	32	0.31	0.11	0.868
Subdural hemorrhage	33	0.47	0.244	0.903
Gastric ulcer, chronic or unspecified with hemorrhage	34	1.403	1.103	1.784
Calculus of ureter	35	0.621	0.403	0.959
Necrotizing fasciitis	36	1.591	1.052	2.407
Fracture of intertrochanteric section of femur, closed	37	1.285	1.026	1.61

## Discussion

With better medical quality and living condition, geriatric patient population is growing and often pose a significant challenge in surgery and anesthesia. Geriatric patients are relative fragile and also develop more complication after anesthesia than general population [1, 15]. The most common postoperative complication is pulmonary complication and the secondary is cardiac event, leading to longer hospitalization and increased mortality. In previous study in Taiwan, relationship between postoperative complications and mortality risk was established, but there was no analysis between preoperative comorbidities and post-operative mortality. The leading preoperative comorbidities were listed as following: Hypertension, Diabetes mellitus, Coronary artery disease, Pulmonary disease, Malignancy, Hepatic dysfunction, and Renal dysfunction. Detailed evaluation and better communicating the aforementioned risk factors to these patients before operation are suggested for improving anesthesia quality and surgical outcomes [16].

A comprehensive geriatric assessment including Activities of Daily Living (IADL), cognitive function, nutrition status, and past medical history were used to predict postoperative morbidity and mortality in geriatric patients who received elective surgery [17, 18]. They came to a conclusion that aging itself not increase surgical risk, rather, the increasing prevalence of chronic disease and the deterioration of the organ’s functions, might increase the risk of postoperative mortality. Geriatric patients tend to carry more than one comorbidity and it is a risk factor for functional decline, disability, dependency, and institutionalization. Risk of functional decline and deterioration of the organ’s functions following comorbidities rather than age itself play an more important role in geriatric patients surgical risk assessment.

In 2015, several large scale study concerning postoperative morbidity and mortality were published, including using multidimensional frailty score to predict postoperative complications in older female cancer patients [18], peer review reporting ASA classification as a reliable independent predictor of medical complications and mortality following surgery [7], a retrospective cohort study using national anesthesia clinical outcome registry [19] on perioperative mortality in 2010 to 2014, the effect of adding functional classification to ASA status for predicting 30-day mortality [20], and newly established preoperative score to predict postoperative mortality (POSPOM) [21]. All the above indicate that the lacking and desiring of an objective preoperative evaluation tool to predict perioperative risk and morbidity.

This is the first retrospective cohort study investigating relationship of comorbidity of elder patients with 30 day post-anesthetic mortality rate using International Classification of Diseases, 9th Revision, Clinical Modification (ICD-9-CM) from Taiwan Health Insurance database. We solely investigated disease code in our study to diminish other man-made bias in the health insurance database and aggregated them into 73 comorbidities by expertise to include most comorbidities. We also adopted death date to include both in-hospital and out-of-hospital death to avoid mortality bias. We used 1:4 propensity score matching case control to select comparable controls, but there were still significant differences in age and sex proportions (*p* < 0.001). A possible explanation is that the large sample size in the present study might be the reason for the statistical significance, but not clinically significant [22]. For example, the difference between 76 years old in non-death group and 78 years old in death group. Multivariate logistic model with stepwise variable selection was then applied to analysis the ability of comorbidities to predict postoperative mortality. From the 33 comorbidities, the leading comorbidity predicts post-anesthetic mortality in order is chronic renal failure, acute myocardial infarction, and intracerebral hemorrhage.

In the past, cardiovascular disease was regarded as the leading comorbidity that contribute to aged patients’ functional decline [23]. Due to poor cardiopulmonary reserve, limited daily activity and function capacity resulted in disability and institutionalization. However, chronic renal dysfunction was found to have better predicting ability to postoperative mortality than myocardia infarction by stepwise variable selection in our study. This can be explained by the increasing number of hemodialyzed patients in Taiwan after National Health Insurance put into practice. Due to low cost of insurance fee, patients with chronic renal failure received more medical care and have longer life span. However, multiple organ system deteriorated rapidly and thromboembolic events increased with longer duration of hemodialysis [24]. Amputation and artificial vascular surgery put these patients in a higher mortality rate after anesthesia [25]. Chronic kidney disease associated with increased risk of death, increased cardiovascular events and hospitalization was proven [26] and it also increased adverse outcome after elective orthopedic, general, and vascular surgery [27].

The secondary leading comorbidity predicting post-anesthetic mortality was acute myocardial infarction compatible as other studies. Risks related to the patient and related to surgery are both high for unstable hemodynamic status and emergent coronary artery bypass. A recent myocardial infarction remains a significant risk factor for postoperative MI and mortality and postponing elective operation after optimizing medical treatment is suggested [28]. Intracerebral hemorrhage was the tertiary leading comorbidity which is correlated with hemorrhagic stroke and traumatic injury accompany with poor outcome. Intracerebral hemorrhage is the most devastating type of stroke leading to greatest mortality and it is also an important public health problem leading to high rates of disability in geriatric patients [29]. Post-operative mortality is high in patients diagnosed as intracerebral hemorrhage undergoing blood evacuation.

In Current era of informative age, large scale of medical data was stored and established as a database in the national health insurance institute. From that, enormous amount of information can be acquired and work up. The limitation of our study is that our database is 1 million Longitudinal Health Insurance Database in 2005. The population is small and the data is old. The international classification of disease(ICD-9) had revised to 10th version and aggregation of ICD-9 codes made man-made bias. Besides, functional classification of ASA and geriatric dysfunction assessment were not included in the database of National Taiwan Health Insurance. Better registration system and further studies were warranted.

## Conclusions

We design this study to present the leading comorbidity contributing to the postoperative mortality in elderly patients in Taiwan from Taiwan’s National Health Insurance Database. In our study, we diminish the impact of type of surgery, age, and sex by using matched propensity score and we use death date as the definition of mortality, which include in-hospital and out-of-hospital mortality. We concluded that chronic renal failure, acute myocardial infarction, and intracerebral hemorrhage are the leading comorbidity contribute to post-anesthetic mortality in geriatric patients in Taiwan. Our findings highlight the clinical importance of chronic renal failure in geriatric population.

## Additional file

Additional file 1: Original and operating code. We aggregated original codes into disease group to resemble clinical pre-anesthetic usage, and called it operating code. This process was conducted independently by three anesthesiologists. (DOCX 78 kb)
