# Supplementary material for: Leading Comorbidity associated with 30-day post-anesthetic mortality in geriatric surgical patients in Taiwan: a retrospective study from the health insurance data
Source: BMC Geriatr. 2017 Oct 24;17:245. doi: 10.1186/s12877-017-0629-7 (PMC5654003; doi:10.1186/s12877-017-0629-7)
Supplement: Additional file 1: — Original and operating code. We aggregated original codes into disease group to resemble clinical pre-anesthetic usage, and called it operating code. This process was conducted independently by three anesthesiologists. (DOCX 78 kb) [file 12877_2017_629_MOESM1_ESM.docx]

Operating coding Original coding

Ischemic heart disease

Myocardial infarction

410 410 *Acute myocardial infarction

41071 Acute subendocardial infarction, initial episode of care

41091 Acute myocardial infarction of unspecified site

414 41401 Coronary atherosclerosis of native coronary art

Hypertension

Systemic hypertension

401 401 *Essential hypertension

4019 Essential hypertension, unspecified

Heart failure

428 428 *Heart failure

4280 Congestive heart failure

785 78551 Cardiogenic shock

Vascular disease

Peripheral arterial occlusive disease

444 44422 Arterial embolism and thrombosis of lower extremity

785 7854 Gangrene

Respiratory disease

Pneumonia

486 486 Pneumonia, organism unspecified

507 5070 Pneumonitis due to inhalation of food or vomitus

510 5109 Empyema, without mention of fistula

Chronic obstructive pulmonary disease

491 491 *Chronic bronchitis

49121 Obstructive chronic bronchitis with acute exacerbation

Restrictive lung disease

511 5119 Pleurisy, unspecified pleural effusion

Respiratory failure

518 5185 Pulmonary insufficiency following trauma and surgery

51881 Acute respiratory failure

Disease of liver and biliary tract

452 45260 Esophageal varices with bleeding

Cirrhosis

571 571 *Chronic liver disease and cirrhosis

5712 Alcoholic cirrhosis of liver

5715 Cirrhosis of liver without mention of alcohol

Disease of GI system

531 53140 Gastric ulcer, chronic or unspecified with hemorrhage, without mention of obstruction

557 5570 Acute vascular insufficiency of intestine

5579 Unspecified vascular insufficiency of intestine

560 56081 Intestinal or peritoneal adhesions with obstruction(postoperative)(postinfection)

Acute pancreatitis

578 5789 Hemorrhage of gastrointestinal tract, unspecifi

Peritonitis

531 53150 Gastric ulcer, chronic or unspecified with perforation, without mention of obstruction

532 53250 Duodenal ulcer, chronic or unspecified with perforation, without mention of obstruction

533 53350 Peptic ulcer, site unspecified, chronic or unspecified with perforation, without mention of obstruction

540 5400 Acute appendicitis, with generalized peritonitis

5409 Acute appendictis, without mention of peritonit

567 567 *Peritonitis

5679 Unspecified peritonitis

569 56983 Perforation of intestine

Urinary disease

162 1623 Tuberculosis of ureter, tubercle bacilli found

1625 Tuberculosis of ureter, tubercle bacilli not fo

403 40391 Unspecified hypertensive renal disease with renal failure

Acute renal failure

584 584 *Acute renal failure

5849 Acute renal failure, unspecified

Chronic renal failure

585 585 Chronic renal failure

591 Hydronephrosis

592 5921 Calculus of ureter

599 5990 Urinary tract infection, site not specified

5997 Hematuria

600 6000 Hypertrophy (benign) of prostate

Endocrine disease

Diabetes mellitus

250 250 *Diabetes mellitus

25000 Diabetes mellitus without mention of complication, Type II [non-insulin dependent type][NIDDM type] [ adult-onset type] or unspecified type, not stated as uncontrolled

2501 *Diabetes with ketoacidosis

25070 Diabetes with peripheral circulatory disorders, Type II [non-insulin dependent type][NIDDM type][adult-onset type] or unspecified type ,not stated as uncontrolled

25080 Diabetes with other specified manifestations, Type II [non-insulin dependent type][NIDDM type][adult-onset type] or unspecified type, not stated as uncontrolled

Musculoskeletal disease

707 7070 Decubitus ulcer

724 72402 Spinal stenosis, lumbar region

733 73313 Pathologic fracture of vertebrae

820 82021Fracture of intertrochanteric section of femur, closed

8208 Fracture of unspecified part of neck of femur, closed

Sepsis

389 389 Unspecified septicemia

728 72886 Necrotizing fasciitis

790 7907 Bacteremia

CVA or trauma

331 3314 Obstructive hydrocephalus

348 3488 Other conditions of brain

Cerebrovascular accident

430 Subarachnoid hemorrhage

431 Intracerebral hemorrhage

432 4321 Subdural hemorrhage

434 43491 Unspecified cerebral artery occlusion with cerebral infarction

785 78559 Other shock without mention of trauma

851 85185 Other and unspecified cerebral laceration

852 85205 Subarachnoid hemorrhage following injury withou

85225 Subdural hemorrhage following injury without mention of open intracranial wound,with prolonged (more than 24 hours) loss of consciousness w/o return to pre-existing consc

85226 Subdural hemorrhage following injury without mention of open intracranial wound, with loss of consciousness of unspecified duration

853 85305 Other and unspecified intracranial hemorrhage f

800 80025 Fracture of vault of skull, closed with subarch

801 80125 Fracture of base of skull, closed with subarchn

Cancer

141 1419 Malignant neoplasm of tongue, unspecified

145 1450 Malignant neoplasm of cheek mucosa

1459 Malignant neoplasm of mouth, unspecified

147 1479 Malignant neoplasm of nasopharynx, unspecified

148 1489 Malignant neoplasm of hypopharynx, unspecified

150 1503 Malignant neoplasm of upper third of esophagus

1505 Malignant neoplasm of lower third of esophagus

1508 Malignant neoplasm of other specified part of e

1509 Malignant neoplasm of esophagus, unspecified

151 1512 Malignant neoplasm of pyloric antrum of stomach

1519 Malignant neoplasm of stomach, unspecified

153 1533 Malignant neoplasm of sigmoid colon

1536 Malignant neoplasm of ascending colon

154 1540 Malignant neoplasm of rectosigmoid junction

1541 Malignant neoplasm of rectum

155 1550 Malignant neoplasm of liver, primary

1551 Malignant neoplasm of intrahepatic bile ducts

157 1570 Malignant neoplasm of head of pancreas

162 1623 Malignant neoplasm of upper lobe, bronchus or lung

1625 Malignant neoplasm of lower lobe, bronchus or lung

1629 Malignant neoplasm of bronchus and lung, unspec

174 1749 Malignant neoplasm of female breast, unspecifie

180 1809 Malignant neoplasm of cervix uteri, unspecified

183 1830 Malignant neoplasm of ovary

185 185 Malignant neoplasm of prostate

188 1889 Malignant neoplasm of bladder, part unspecified

196 1960 Secondary and unspecified malignant neoplasm of lymph nodes of head, face, and neck

1962 Secondary and unspecified malignant neoplasm of

197 1970 Secondary malignant neoplasm of lung

1972 Secondary malignant neoplasm of pleura

1976 Malignant neoplasm of retroperitoneum and perit

1977 Secondary malignant neoplasm of liver

198 1982 Secondary malignant neoplasm of skin

1983 Secondary malignant neoplasm of brain and spinal cord

1985 Secondary malignant neoplasm of bone and bone m

19889 Secondary malignant neoplasm of other specified sites

Others

V581 V581 Encounter for chemotherapy

996 9961 Mechanical complication of other vascular device, implant and graft

99662 Infection and inflammatory reaction due to other vascular device, implant, and graft

99673 Other complications due to renal dialysis device, implant, and graft

332 332 Parkinsonism
